# Inflammatory biomarkers, disease activity and spinal disease measures in patients with ankylosing spondylitis after treatment with infliximab

**DOI:** 10.1136/ard.2007.071605

**Published:** 2007-07-20

**Authors:** S Visvanathan, C Wagner, J C Marini, D Baker, T Gathany, J Han, D van der Heijde, J Braun

**Affiliations:** 1 Centocor Research and Development, Inc., Malvern, PA, USA; 2 Department of Rheumatology, Leiden University Medical Centre, The Netherlands; 3 Rheumazentrum-Ruhrgebiet, Herne, Germany

## Abstract

**Objective:**

To evaluate the relationship between biomarker levels and disease activity and the spinal inflammation detected by magnetic resonance imaging (MRI) in patients with ankylosing spondylitis (AS).

**Methods:**

Patients with AS were randomly assigned in a 3:8 ratio to receive infusions of placebo or 5 mg/kg infliximab at weeks 0, 2, 6, 12 and 18. Sera were collected for biomarker analysis at weeks 0, 2 and 24 and were analysed for levels of interleukin-6 (IL-6), vascular endothelial growth factor (VEGF) and C-reactive protein (CRP). Bath Ankylosing Spondylitis Disease Activity Index (BASDAI) scores and pre- and post-gadolinium T1 and short τ inversion recovery MRIs were collected at baseline and week 24.

**Results:**

Significantly greater reductions in IL-6, VEGF and CRP were observed at weeks 2 and 24 in the infliximab group compared with the placebo group (all p<0.001). Baseline IL-6 levels >7.38 pg/ml and CRP levels >1.5 mg/dl were associated with increased rates of clinical response after 24 weeks. Multiple regression analyses showed that reductions from baseline to week 2 in IL-6, but not CRP or VEGF, were significantly associated with reductions in MRI activity and BASDAI scores from baseline to week 24 in the infliximab group (p<0.001).

**Conclusions:**

Significant reductions in IL-6, VEGF and CRP were observed with infliximab compared with placebo. High levels of baseline IL-6 and CRP were associated with clinical response after infliximab treatment. Early reductions in IL-6 were significantly associated with improvements in disease activity and the spinal inflammation detected by MRI.

Ankylosing spondylitis (AS) is associated with persistent inflammation of the sacroiliac joints and spine, which can lead to bone erosions, syndesmophytes or complete ankylosis of the spine. There is evidence that the inflammation in AS is at least partly mediated by tumour necrosis factor (TNF)-α and interleukin (IL)-6, as high levels of these cytokines have been found in biopsies from the sacroiliac joints of patients with AS.^1^ ^2^ Elevated serum levels of TNF-α and vascular endothelial growth factor (VEGF) have been shown to be correlated with disease activity and C-reactive protein (CRP) levels.^3^ ^4^

VEGF is an important regulator of angiogenesis, which is key to the inflammatory process. Polymorphisms in the VEGF genes were reported to be associated with disease severity in patients with AS.^5^ ^6^

IL-6 is a multifunctional cytokine that regulates immune response, haematopoiesis, acute phase response and inflammation. Dysregulation of IL-6 production is implicated in the pathology of several disease processes,^7^^–^9 and increased IL-6 levels have been associated with rheumatoid arthritis, systemic-onset juvenile chronic arthritis, osteoporosis and psoriasis.^10^^–^12 IL-6 is critically involved in experimentally induced autoimmune disease, such as antigen-induced arthritis, and experimental allergic encephalomyelitis.^13^ ^14^ Collectively, these data from both clinical studies and animal models suggest that IL-6 plays a critical role in the pathogenesis of immune-mediated diseases,^15^ supporting the face validity of the use of biomarkers to predict treatment response.

The course of spinal inflammation associated with AS can be demonstrated by magnetic resonance imaging (MRI).^16^ ^17^ While a significant reduction in spinal inflammation has been shown with MRI after treatment with anti-TNF-α agents,^18^^–^21 a direct link between clinical disease activity and spinal inflammation as seen on MRI and osteoproliferative changes has not been well characterised.

In the current study, biomarkers known to be important in the inflammatory and angiogenic processes that occur in AS were selected for evaluation over time. The first aim of this study was to determine whether treatment with infliximab modulates specific biomarkers in patients with AS, and when such modulation may occur. The second aim was to evaluate associations between changes in biomarker levels and disease activity and inflammation detected by MRI.

## MATERIALS AND METHODS

The details of the ASSERT (Ankylosing Spondylitis Study for the Evaluation of Recombinant Infliximab Therapy) study have been published previously.^22^ Sera from patients who received placebo or 5 mg/kg infliximab were collected for biomarker analysis at weeks 0, 2, and 24. IL-6, VEGF and interferon (IFN)-γ were evaluated as markers of inflammation. Enzyme-linked immunoabsorbent assay kits for IL-6 and VEGF were purchased from R&D Systems (Minneapolis, MN, USA), and those for IFN-γ were purchased from Biosource, Europe S.A. The Tina-quant kit from Roche (Indianapolis, IN, USA) was used to determine CRP levels. All assays were validated by scientists at Clinical Pharmacology and Experimental Medicine, Centocor Research and Development, Inc. before use on study samples. Biomarker levels that were below the lower limit of quantification (LLOQ) were considered to be undetectable.

Disease activity was assessed using the Bath Ankylosing Spondylitis Disease Activity Index (BASDAI), which has a possible score of 0–10 with a higher score indicating greater disease activity.^23^ Clinical response was assessed by determining the number of patients who achieved a 50% improvement in the BASDAI score (BASDAI 50) and the number of patients who achieved 20% improvement in the ASsessment in Ankylosing Spondylitis working group criteria (ASAS 20).^24^

MRI was conducted at baseline and week 24 as previously reported.^20^ Pre- and post-gadolinium T1 and short τ inversion recovery magnetic resonance images of the spine were acquired at baseline and week 24. The activity score for each vertebral unit ranged from 0 to 6, and the total activity score for the spine ranged from 0 to 138 (23 vertebral units from C2 to S1).^19^ ^25^^–^27

### Statistical analyses

To validate the use of biomarkers in predicting the treatment response in patients with AS who received infliximab, we evaluated the face validity, discriminant capacity, predictive validity and sensitivity to change. Face validity was determined by assessing the relationship between biomarker levels and disease activity at baseline. Discriminant capacity and sensitivity to change was assessed by demonstrating the effect of infliximab treatment on biomarker levels relative to placebo after 2 and 24 weeks of follow-up. Predictive validity was evaluated by determining the association between changes from baseline in disease activity (BASDAI and MRI scores) and biomarker levels at baseline, week 2 and week 24.

The median percentage change from baseline for each biomarker was determined at weeks 2 and 24. Statistical comparisons were made between the placebo and infliximab groups using an analysis of variance on the van der Waerden scores. Biomarker data for patients with sample baseline values less than the LLOQ were excluded from analyses.

Univariate Spearman Rank correlations were computed to determine the relationship between baseline levels of IL-6, VEGF and CRP. Correlation analyses were also used to examine relationships between (1) the change from baseline to week 24 in MRI activity score and the biomarker levels (baseline and changes from baseline to week 2 and week 24), and (2) the associations between change from baseline to week 24 in BASDAI and biomarker levels (baseline and changes from baseline to week 2 and week 24).

To further characterise the relationship between biomarker levels, disease activity and inflammation at baseline, patients were categorised into quantiles of baseline CRP and IL-6, and descriptive statistics of baseline MRI activity and BASDAI scores were calculated for each category. For CRP, patients were divided into tertile categories (<0.9 mg/dl, ⩾0.9 and <2.4 mg/dl, or ⩾2.4 mg/dl) because all patients had detectable levels of CRP at baseline (table 1). Nearly half of the patients were assigned the same baseline IL-6 value (3.13 pg/ml, which was one-half of the LLOQ of 6.25 pg/ml) because their levels were below the LLOQ (table 1). Therefore, patients were divided into categories of IL-6 levels based on the median IL-6 value at baseline (<7.38 pg/ml or >7.38 pg/ml). The percentage of patients who achieved an ASAS 20 or BASDAI 50 response at week 24 was also calculated for each category of baseline CRP and IL-6.

**Table 1:** Baseline characteristics

Assessment	Placebo n = 78	5 mg/kg Infliximab n = 201
Men, no. (%)	68 (87.2)	157 (78.1)
Disease duration, years		
n	76	201
Mean (SD)	11.9 (8.0)	10.1 (8.7)
Median (IQ range)	13.2 (3.7, 17.9)	7.7 (3.3, 14.9)
HLA-B27 positive		
n	78	200
No. (%)	69 (88.5)	173 (86.5)
Inflammation (average morning stiffness on a visual analogue scale 0–10 cm)		
n	78	201
Mean (SD)	6.9 (1.9)	6.9 (2.3)
Median (IQ range)	7.0 (5.7, 8.3)	7.3 (5.4, 8.5)
BASDAI score		
n	78	201
Mean (SD)	6.2 (1.6)	6.5 (1.5)
Median (IQ range)	6.5 (5.2, 7.1)	6.6 (5.3, 7.6)
C-reactive protein (mg/dl)		
n	78	201
No. (%) with values ⩾LLOQ	78 (100)	201 (100)
Mean (SD)	2.4 (2.8)	2.4 (2.7)
Median (IQ range)	1.7 (0.7, 3.3)	1.5 (0.7, 3.2)
Interleukin-6 (pg/ml)		
n	67	187
No. (%) with values ⩾LLOQ	32 (47.8)	105 (56.1)
Mean (SD)	11.4 (17.2)	13.2 (20.0)
Median (IQ range)	3.1 (3.1, 14.8)	7.7 (3.1, 14.2)
Vascular endothelial growth factor (pg/ml)		
n	75	193
No. (%) with values ⩾LLOQ	75 (100.0)	191 (99.0)
Mean (SD)	556.2 (385.6)	520.4 (361.5)
Median (IQ range)	473.5 (289.5, 738.0)	421.5 (279.9, 664.3)
Interferon-γ (pg/ml)		
n	62	165
No. (%) with values ⩾LLOQ	3 (4.8)	9 (5.5)
Mean (SD)	0.7 (1.3)	0.6 (0.3)
Median (IQ range)	0.5 (0.5, 0.5)	0.5 (0.5, 0.5)

BASDAI, Bath Ankylosing Spondylitis Disease Activity Index; IQ, interquartile; LLOQ, lower limit of quantification.

A multiple linear regression analysis was performed to explore baseline levels of IL-6, CRP and VEGF as potential indicators of the change from baseline to week 24 in MRI activity scores and the change from baseline to week 24 in BASDAI scores. Similar analyses were performed to evaluate the percentage changes in these biomarkers from baseline to week 2 and from baseline to week 24.

Statistical analyses were performed using the SAS system, version 8.2 (SAS Institute, Cary, NC, USA). P-values have been provided for exploratory purposes and were not adjusted for multiplicity. Statistical tests were two-sided, and p<0.05 was considered significant.

## RESULTS

### Baseline characteristics and biomarker levels

The baseline characteristics of patients in the ASSERT trial are summarised in table 1. The study population was typical of patients with well-established, active AS. As reported previously,^22^ the majority of the patients in ASSERT were men, most of whom tested positive for the HLA-B27 allele. The median assessment of average morning stiffness on a visual analogue scale was 7.0 in the placebo group and 7.3 in the infliximab group. Although there appeared to be a disparity between the groups in the median disease duration (13.2 years in the placebo group and 7.7 years in the infliximab group), the means were similar (11.9 and 10.1 years, respectively).

The treatment groups were also generally comparable for all biomarkers of inflammation at baseline. The median IL-6 level in the placebo group (3.1 pg/ml) was lower than that of the infliximab group (7.7 pg/ml), but the means were comparable (11.4 vs 13.2, respectively). Approximately half of the patients had IL-6 levels that were above the LLOQ for the assay (47.8% and 56.1% of placebo and infliximab groups, respectively). Only 12 patients had IFN-γ levels that were above the LLOQ for the assay (4.8% and 5.5%, respectively).

### Correlations between individual biomarker levels at baseline and after treatment with infliximab or placebo

In all patients, Spearman Rank correlation coefficients among baseline biomarker levels showed a strong and statistically significant relationship between IL-6 and CRP levels (0.698, p<0.001). Moderate correlations between CRP and VEGF (0.362, p<0.001) and IL-6 and VEGF (0.264, p<0.001) were also observed.

Correlations among changes in biomarker levels at week 24 were also evaluated. In the infliximab group, there were significant relationships between change from baseline in IL-6 and CRP (r = 0.689, p<0.001), IL-6 and VEGF (r = 0.445, p<0.001), and VEGF and CRP (r = 0.565, p<0.001) at week 24. In the placebo group, only a significant correlation between IL-6 and CRP was observed (r = 0.452, p<0.001).

### Face validity: baseline biomarker levels and disease activity, peripheral involvement and extra-articular manifestations

At baseline, Spearman correlation coefficients between BASDAI scores and IL-6 levels (0.134, p = 0.0327) and BASDAI scores and CRP levels (0.167, p = 0.005) were similar, as were those between MRI activity scores and IL-6 levels (0.240, p = 0.0001) and MRI activity scores and CRP levels (0.282, p<0.0001).

**Figure 1:**
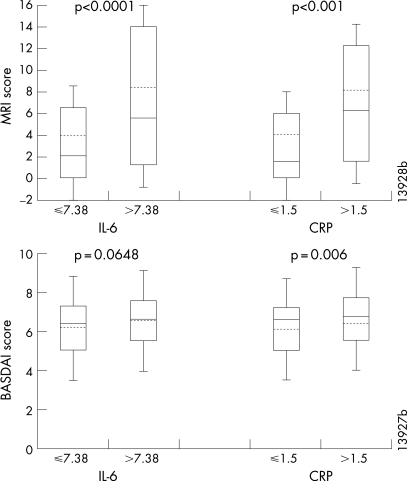
Baseline magnetic resonance imaging (MRI) activity and BASDAI (Bath Ankylosing Spondylitis Disease Activity Index) scores for quantiles of baseline interleukin (IL)-6 and C-reactive protein (CRP). Horizontal solid lines are medians, and horizontal dotted lines are means. The boxes show the interquartile ranges. The error bars show the standard deviations. P-values show the difference between quantiles of each biomarker using an analysis of variance on the van der Waerden normal scores.

We also evaluated baseline MRI activity and BASDAI scores for subgroups of baseline IL-6 and CRP (fig 1). Patients with IL-6 levels greater than the median (7.38 pg/ml, hereafter referred to as patients with elevated IL-6) had significantly greater MRI activity scores than those with IL-6 levels at or below the median (p<0.001). Overall, 75.0% of patients with elevated IL-6 had a baseline MRI activity score >1 compared with 56.3% of patients with low IL-6 (p = 0.002). Similarly, patients with CRP levels >3 times the upper limit of normal (normal range 0–0.5 mg/dl) had significantly greater MRI activity scores compared with those with CRP levels ⩽3 times the upper limit of normal (p<0.001). The trends between baseline IL-6 or CRP levels and BASDAI scores were similar to those observed for MRI activity scores; however, the magnitude of the differences between the medians of the groups was not as great for BASDAI as it was for the MRI activity scores (p = 0.0648 for IL-6 and p = 0.006 for CRP) (fig 1).

Patients with elevated IL-6 levels at baseline were also more likely to have peripheral joint involvement than those with low IL-6 levels (50.0% vs. 28.1%, respectively, p<0.001) and had a greater mean (SD) number of peripheral swollen joints (2.02 (3.44) joints versus 1.20 (3.30) joints, p<0.001). Moreover, 78.9% of patients with elevated IL-6 levels had CRP levels >3 times the upper limit of normal compared with 17.2% of patients with low IL-6 levels (p<0.001). The baseline mean (SD) CRP level for patients with elevated IL-6 levels was 3.79 (3.27) mg/dl compared with 1.04 (0.81) mg/dl for patients with low IL-6 levels (p<0.001). There were no statistically significant differences in age, disease duration, baseline inflammation (visual analogue scale), or the presence of uveitis between patients with low and high IL-6 levels (data not shown).

### Discriminant capacity and sensitivity to change: reduction in biomarker levels after treatment with infliximab or placebo

**Figure 2:**
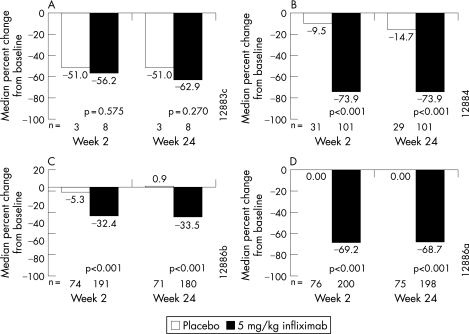
Median percentage change from baseline in (A) interferon-γ, (B) interleukin-6, (C) vascular endothelial growth factor and (D) C-reactive protein.

Percentage changes in biomarker levels from baseline to week 2 and from baseline to week 24 are shown for infliximab- and placebo-treated patients in fig 2. Significantly greater reductions in IL-6, VEGF and CRP levels were observed at both week 2 and week 24 for patients in the infliximab group compared with the placebo group (p<0.001). There was no difference between the treatment groups in the change in IFN-γ levels.

### Predictive validity: correlations between biomarker levels and changes in disease activity and spinal inflammation

Univariate correlations between biomarker levels and changes in MRI activity and BASDAI scores are summarised in table 2. In the infliximab group, baseline IL-6 and CRP levels were significantly correlated inversely with changes from baseline to week 24 in MRI activity scores (p⩽0.001) and BASDAI scores (p⩽0.001), with high baseline IL-6 and CRP levels associated with greater reductions in the two indices. In addition, percentage changes from baseline to week 2 and 24 in IL-6, VEGF and CRP levels were significantly correlated with changes in MRI activity scores (p<0.05) and BASDAI scores (p⩽0.001) from baseline to week 24. Thus, decreases from baseline to week 2 and 24 in IL-6, CRP and VEGF levels were significantly correlated with improvement in MRI activity and BASDAI scores. Correlations with changes in IFN-γ levels were not evaluated because of the small number of patients who had detectable IFN-γ levels at baseline.

**Table 2:** Univariate correlations between biomarker levels (baseline and percentage change from baseline to week 2 and week 24) and change from baseline to week 24 in disease activity (BASDAI) and the inflammation detected by MRI (MRI activity score) in patients with ankylosing spondylitis who received infliximab 5 mg/kg (n = 201) or placebo (n = 78)

Biomarker	Change in MRI activity score		Change in BASDAI	
	Placebo	Infliximab	Placebo	Infliximab
Baseline				
IL-6	−0.158	−0.205***	0.062	−0.258***
VEGF	0.067	−0.074	0.140	−0.079
CRP	−0.047	−0.291***	0.169	−0.322***
Percentage change from baseline to week 2				
IL-6	0.080	0.260***	0.066	0.297***
VEGF	0.110	0.243***	0.105	0.260***
CRP	0.006	0.296***	−0.017	0.347***
Percentage change from baseline to week 24				
IL-6	−0.086	0.215**	0.178	0.340***
VEGF	−0.101	0.170*	0.148	0.330***
CRP	−0.090	0.243***	0.147	0.414***

*p<0.05.

**p⩽0.01.

***p⩽0.001.

BASDAI, Bath Ankylosing Spondylitis Disease Activity Index; CRP, C-reactive protein; IL-6, interleukin-6; MRI, magnetic resonance imaging, VEGF, vascular endothelial growth factor.

### Predictive validity: baseline biomarker levels and ASAS 20 and BASDAI 50 response

**Figure 3:**
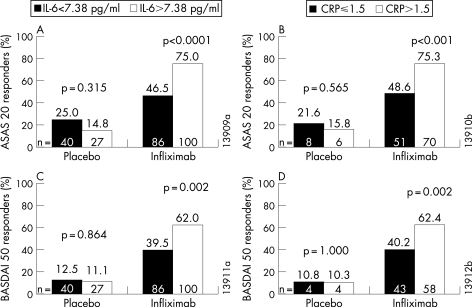
Comparison of the percentage of ASAS 20 (20% improvement in the ASsessment in Ankylosing Spondylitis working group criteria) (A,B) and BASDAI (Bath Ankylosing Spondylitis Disease Activity Index) 50 (C,D) responders between categories of baseline interleukin (IL)-6 and C-reactive protein (CRP) divided at the medians.

Figure 3 shows the treatment response of patient groups categorised according to their baseline biomarker levels. Among patients who received infliximab, a greater percentage of those with elevated IL-6 at baseline (>7.38 pg/ml) demonstrated a clinical response as measured by ASAS 20 (A, p<0.0001) and BASDAI 50 (C, p = 0.002) compared with those with low IL-6. Similarly, a greater percentage of infliximab-treated patients with CRP >3 times the upper limit of normal (1.5 mg/dl) achieved an ASAS 20 (B, p<0.001) and BASDAI 50 (D, p = 0.002) responses compared with the percentage with CRP ⩽3 times the upper limit of normal.

### Predictive validity: multiple regression associations between biomarker levels and changes in disease activity and spinal inflammation

Multiple linear regression analyses were conducted to evaluate whether one individual marker (IL-6, VEGF and CRP) was more strongly associated with change in MRI activity or BASDAI scores from baseline to week 24 (table 3). Early reductions in IL-6 from baseline to week 2 were significantly associated with improvement from baseline to week 24 in MRI activity and BASDAI. Reductions from baseline to week 24 in CRP were significantly associated with reductions in MRI activity scores at week 24; however, both baseline levels and the decrease in CRP from baseline to week 24 were associated with reductions in BASDAI. Further, reductions in VEGF levels at week 24 were also significantly associated with change from baseline to week 24 in BASDAI. Thus, in the infliximab group, early decreases in IL-6 and later decreases in CRP were associated with improvement in MRI activity scores. Further, early decreases in IL-6 and baseline CRP levels were associated with improvement in BASDAI scores.

**Table 3:** Linear regression modelling of the associations between biomarker levels (baseline and percentage change from baseline to week 2 or week 24) and change from baseline to week 24 in disease activity (BASDAI) and the spinal inflammation detected by MRI (MRI activity score)

Model	r^2^	b	p Value
Infliximab group			
MRI activity score: change from baseline to week 24			
Baseline biomarker levels			
no parameters significantly associated	−	−	−
Percentage change from baseline to week 2 in biomarker levels	0.011		
IL-6		0.053	<0.001
Percentage change from baseline to week 24 in biomarker levels	0.042		
CRP		0.009	0.009
BASDAI: change from baseline to week 24			
Baseline biomarker levels	0.043		
CRP		−0.196	0.005
Percentage change from baseline to week 2 in biomarker levels	0.099		
IL-6		0.021	<0.001
Percentage change from baseline to week 24 in biomarker levels	0.113		
VEGF		0.005	0.029
CRP		0.005	0.005
Placebo group			
No biomarker levels at baseline or changes from baseline to week 2 or 24 were significantly associated with changes in MRI activity score or BASDAI at week 24			

BASDAI, Bath Ankylosing Spondylitis Disease Activity Index; CRP, C-reactive protein; IL-6, interleukin-6; MRI, magnetic resonance imaging; VEGF, vascular endothelial growth factor.

## DISCUSSION

The results of this study show that the established efficacy of infliximab for improving the clinical signs and symptoms of AS and reducing spinal inflammation demonstrated by MRI^19^^–^21 is also associated with significant changes in inflammatory biomarkers. Understanding the utility of specific biomarkers associated with anti-TNF-α therapy may be particularly important in AS because the rate of structural changes is rather slow over time,^28^ ^29^ and early identification of patients who will benefit from therapy is essential. The available information on the modulation of inflammatory markers in AS after treatment with anti-TNF agents and the association with changes in imaging measures such as MRI has been limited. Data from an early randomised controlled trial^30^ ^31^ and a more recent open-label study in a small number of patients^32^ suggest that baseline CRP levels may be useful as markers of clinical response after treatment with infliximab. The results of the current study represent, to our knowledge, the largest data set of patients with AS evaluated for inflammatory markers, clinical signs and symptoms, and the inflammation detected by MRI.

The face validity of inflammatory marker levels was already evident at baseline. Patients with high baseline IL-6 (>7.38 pg/ml) and CRP (>1.5 mg/dl) had more spinal inflammation detected by MRI compared with those with low biomarker levels. Patients with high baseline CRP levels also had greater disease activity as measured by BASDAI, and there was a trend towards greater disease activity for patients with high baseline IL-6 levels, although the difference was not statistically significant. Although there was variability in MRI activity and BASDAI score within each group, the magnitude of the difference between the groups was greater for MRI activity compared with BASDAI, suggesting that there may be a stronger relationship between biomarker levels and spinal inflammation relative to that between biomarker levels and disease activity.

Following treatment with infliximab, patients showed significantly greater reductions in IL-6, VEGF and CRP compared with patients who received placebo, demonstrating the discriminant capacity and sensitivity to change of the biomarkers. These reductions were evident as early as week 2, and the levels were similarly low at week 24. In line with this result, treatment with infliximab was shown to modulate markers of angiogenesis, including VEGF, in synovial tissue early after initiation of therapy in patients with psoriatic arthritis.^33^

In the infliximab group, baseline IL-6 and CRP levels and reductions from baseline in IL-6, VEGF and CRP were significantly correlated with improvement in disease activity and inflammation at week 24. No significant correlations were observed in the placebo group. The significance of these correlations is noteworthy, because a substantial number of AS patients (about 50%) had undetectable IL-6 levels at baseline. This finding is consistent with the results of an earlier pilot study^34^ and merits further investigation because there is limited knowledge on the natural course of IL-6 levels in AS.

The predictive validity of biomarker levels became particularly evident when the clinical response was evaluated. Among patients who received infliximab, an ASAS 20 and a BASDAI 50 response at week 24 was achieved by a greater percentage of those with elevated IL-6 or CRP levels at baseline compared with those with low IL-6 or CRP levels. The multiple regression analysis enabled us to determine which biomarker was most associated with spinal inflammation and disease activity. The results showed that early changes in IL-6 and late changes in CRP were associated with improvements in both the MRI activity and the BASDAI score. Baseline CRP levels and reductions in VEGF levels at week 24 were also associated with improvements in BASDAI score, but were not associated with changes in MRI activity score. The finding that early improvement in IL-6 was associated with improvement in both spinal inflammation and disease activity suggests that IL-6 may play an earlier and more central role in the inflammatory cascade than CRP. In this regard, it is noteworthy that IL-6 is thought to be involved in the initiation of the acute phase response and the production of CRP in the liver.^35^ IL-6 polymorphisms have been shown to be associated with CRP levels in other conditions.^8^ ^9^

In the current study, baseline levels of IL-6 and CRP were generally comparable between both treatment groups. Levels of serum IL-6 and CRP in previous published studies of patients with AS have been conflicting; one reporting lower levels than those in the current study,^36^ ^37^ and others reporting higher levels.^38^^–^40 The reported differences in IL-6 and CRP levels are most likely related to the heterogeneity of the AS patient populations studied. In the current study, only patients with active disease who fulfilled the criteria for anti-TNF therapy were included. Moreover, IL-6 levels were strongly correlated with CRP levels at baseline in the current study, which is consistent with the results of other studies of AS and rheumatoid arthritis.^38^ ^41^ Moderate correlations were observed between baseline levels of IL-6 and VEGF and between baseline levels of VEGF and CRP. Treatment with infliximab did not alter these relationships between the biomarkers. Baseline VEGF levels in the current study were similar to those previously reported for patients with early rheumatoid arthritis^42^ but were higher than those of other patients with spondyloarthropathies.^4^ In patients with rheumatoid arthritis, IL-6 has been shown to be a key cytokine in the production of VEGF,^43^ and VEGF may, in turn, stimulate the production of IL-6 and TNF-α in a positive feedback loop.^44^ Our results suggest that similar relationships between IL-6 and VEGF may play a part in the inflammation exhibited by some patients with AS. However, as already stated, a significant number of AS patients had undetectable levels of circulating IL-6.

Some potential limitations of this study are noteworthy. The ASSERT study was primarily designed to evaluate the safety and efficacy of infliximab in patients with AS and was not powered to evaluate correlations between changes in inflammatory markers and clinical measures. In addition, the study population was somewhat heterogeneous, which included a range of patients with moderate to severe AS. Finally, the IL-6 assay had limited sensitivity to detect low levels of this cytokine, and thus, nearly half of the patients had undetectable IL-6 levels at baseline. This limitation may have resulted in an underestimation in the strength of the correlations between changes in IL-6 levels and changes in measures of inflammation and disease activity in the infliximab group. However, despite these potential limitations, the results of this study contribute to our understanding of the importance of IL-6 in the pathogenesis of AS. The results suggest that the early reduction of IL-6 may be a critical event in the treatment response and the pathogenesis of AS. Although biomarker levels were associated with improvement in disease activity and the spinal inflammation detected by MRI, their relative contribution to the explanation of the observed treatment response needs to be examined further, especially as there is no strong correlation between BASDAI and MRI (Braun, unpublished). However, the results of this study do suggest that an early reduction of IL-6 level is important. A reduction in CRP level may have utility as a surrogate marker of response in clinical practice, especially given the strong correlation between IL-6 and CRP levels.

MRI has been shown to be a useful tool for evaluating the reduction in spinal inflammation after anti-TNF therapy, but it is unclear how early the benefit can be detected. In any case, the early identification of patients who are most likely to exhibit improvement through the use of biomarkers will assist clinicians in their treatment decisions.
